# Detection of Arsenic(V) by Fluorescence Sensing Based on Chlorin e6-Copper Ion

**DOI:** 10.3390/molecules29051015

**Published:** 2024-02-26

**Authors:** Mao-Ling Luo, Guo-Ying Chen, Jia-Li Wang, Tong-Qing Chai, Zheng-Ming Qian, Wen-Jia Li, Feng-Qing Yang

**Affiliations:** 1 School of Chemistry and Chemical Engineering, Chongqing University, Chongqing 401331, China; 20185486@cqu.edu.cn (M.-L.L.); 20221801017@stu.cqu.edu.cn (G.-Y.C.); 202118021010@cqu.edu.cn (J.-L.W.); 20175531@cqu.edu.cn (T.-Q.C.); 2 Dongguan HEC Cordyceps R&D Co., Ltd., Dongguan 523850, China; qianzhengming@hec.cn

**Keywords:** chlorin e6, fluorescence, copper, arsenic

## Abstract

The high toxicity of arsenic (As) can cause irreversible harm to the environment and human health. In this study, the chlorin e6 (Ce6), which emits fluorescence in the infrared region, was introduced as the luminescence center, and the addition of copper ion (Cu^2+^) and As(V) provoked a regular change in fluorescence at 652 nm, whereas that of As(III) was 665 nm, which was used to optionally detect Cu^2+^, arsenic (As(III), and As(V)). The limit of detection (LOD) values were 0.212 μM, 0.089 ppm, and 1.375 ppb for Cu^2+^, As(III), and As(V), respectively. The developed method can be used to determine Cu^2+^ and arsenic in water and soil with good sensitivity and selectivity. The 1:1 stoichiometry of Ce6 with Cu^2+^ was obtained from the Job plot that was developed from UV–visible spectra. The binding constants for Cu^2+^ and As(V) were established to be 1.248 × 10^5^ M^−1^ and 2.35 × 10^12^ M^−2^, respectively, using B–H (Benesi–Hildebrand) plots. Fluorescence lifetimes, B–H plots, FT–IR, and ^1^H-NMR were used to postulate the mechanism of Cu^2+^ fluorescence quenching and As(V) fluorescence restoration and the interactions of the two ions with the Ce6 molecule.

## 1. Introduction

Arsenic is an abundant and widely distributed non-metallic element, ranking 20th among the elements that make up the earth’s crust [1]. The arsenic exists mainly as arsenite (As(III)) and arsenate (As(V)) in inorganic forms and monomethyl arsenic acid (MMA), dimethyl arsenic acid (DMA), and dithiol arsenate (DTA) in organic forms [2]. The toxicity of inorganic arsenic is much greater than that of organic arsenic, and As(III) is recognized as the most toxic of the inorganic arsenic forms [3]. As(III) can bind to enzymes or proteins containing sulfhydryl (–SH) functional groups in the body, altering their conformation and function and affecting normal physiological processes in the body [4,5]. As(III) and As(V) are structurally similar to phosphite and phosphate ions, which can permanently substitute the phosphate groups to interrupt the transformation of ATP to ADP [5]. Consequently, there are serious health risks associated with chronic exposure to arsenic, such as skin or lung cancer [6], cardiovascular disease [7], and neurological disease [8]. In addition, arsenic and its compounds were officially classified as class 1 carcinogens and specified that arsenic concentrations in drinking water should be controlled at 10.0 μg/L (10 ppb) by the World Health Organization (WHO) in 2017 [9]. Therefore, it is of paramount significance in arsenic content detection for environmental chemistry, life sciences, agriculture, medicine, and other related fields.

Currently, there are many techniques available for copper and arsenic detection, such as atomic absorption/emission spectrometry (AAS/AES) [10,11,12], atomic fluorescence spectroscopy (AFS) [13,14], X-ray fluorescence spectroscopy (XRF) [15,16], inductively coupled plasma–mass spectrometry (ICP–MS) [17,18], electrochemistry [19,20,21], ultraviolet–visible absorption spectrometry (UV–Vis)/colorimetry [22,23,24,25], and fluorescence spectroscopy [26,27,28,29], etc. These methods have their characteristics and advantages for testing different environmental samples, but the drawbacks of some methods, such as AAS/AES and ICP–MS, which require expensive and large instruments and specialized operators, limit their applications [27,30]. Compared to conventional UV analytical methods, fluorescence spectroscopy is more sensitive and presents the advantage of a wider linear range, which is a promising tool for rapid and easy tracking of arsenic in environmental monitoring [5]. In reality, fluorescent probes based on small molecules have been widely used for the detection of arsenic and copper [9,31,32,33,34,35,36,37,38,39,40,41,42,43,44,45,46].

Atoms with N, O, and S in the structure of these ligands can interact with arsenic, resulting in regular changes in fluorescence intensity. For example, Öksüz et al. synthesized a new fluorescent ligand 3′,6′-bis(diethylamino)-2-{[(1E)-(4,5-dimethyl-2furyl)methylene]amino}spiro[isoindole-1,9′-xanthen]-3(2H)-one (DMBD) with good luminescence properties based on rhodamine 6G, in which the carbonyl oxygen can be coordinated to As(III), and the developed fluorescent method is efficient in detecting arsenic in tea leaves with high sensitivity and good accuracy [32]. Similarly, Saha et al. utilized acriflavine as an energy donor and rhodamine B as an energy acceptor for As(V) detection based on fluorescence resonance energy transfer [3]. Lohar et al. synthesized a diformyl-*p*-cresol-based receptor (HL) that can induce chelating fluorescence enhancement (CHEF) through intermolecular H-bonding, and the developed method had a detection limit of 4.1 ppb for As(III) in aqueous solution [35]. Furthermore, Lohar et al. prepared a novel fluorescent probe (APSAL) that can detect intracellular arsenate at the micromolar level using condensation of salicylaldehyde with 4-aminoantipyrine [5].

Different imidazole- and benzimidazole-based fluorescent small-molecule probes have been applied in the copper ion (Cu^2+^) detection by utilizing the specific coordination of Cu^2+^ with nitrogen atoms [40,42,44,45]. Pan et al. designed a probe consisting of triphenylamine as a fluorescent moiety and a benzimidazole derivative as an acceptor for Cu^2+^ detection within 1s [42]. Park et al. synthesized a benzimidazole-based probe BIPMA fluorescence “turn-on” for the detection of Cu^2+^ ions with a low detection limit of 4.80 nM [45]. Furthermore, the introduction of O and S atoms into the molecular probe may increase the selectivity for Cu^2+^. Xie et al. synthesized a rhodamine B-based chemosensor for the fluorescence detection of copper with high affinity and selectivity, and excess EDTA will not interfere with the interaction of Cu^2+^ with this probe [39]. In addition, the benzothiazole-based colorimetric chemosensor BTV synthesized by Heo et al. will not interfered with by other cations in the detection of Cu^2+^ [41]. 

**Figure 1:**
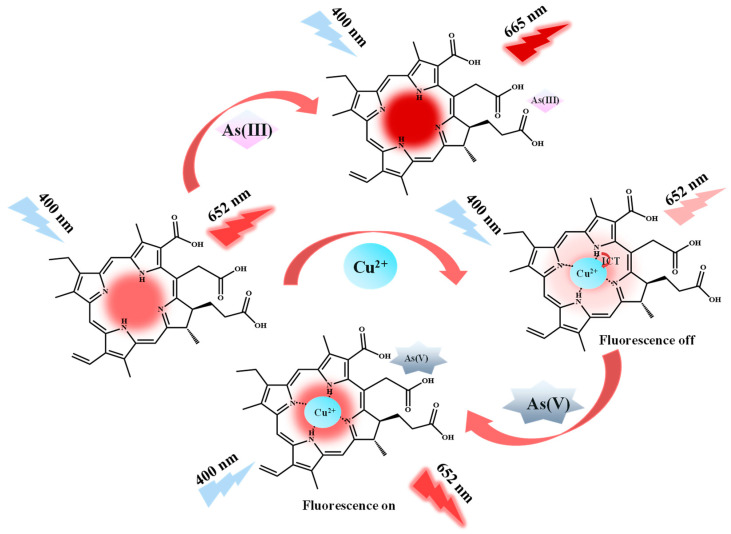
Schematic diagram of determination of As(III), Cu^2+^, and As(V) based on Ce6.

Chlorin e6 (Ce6) (inset of Figure S1) is a chlorophyll degradation product and is commonly used as a photosensitizer in the photodynamic therapy of cancer [47,48]. As a second-generation porphyrin-based photosensitizer, Ce6 is a macrocyclic compound formed by four pyrroles connected by methylene groups, and its porphyrin ring features an expanded conjugated π-electron system and aromatic properties, with the maximum wavelength of excitation and emission at 400 nm and 652 nm in ethanol solution, respectively [49,50]. Moreover, studies by Wang and Patal showed that arsenic can have a significant effect on chlorophyll synthesis and plant growth [51,52]. Therefore, this chlorophyll degradation product (Ce6) was innovatively used for arsenic detection in this study. In brief, the four nitrogen atoms in the porphyrin ring of Ce6 are utilized to provide lone-pair electrons as metal-binding sites for selective coordination with and detection of Cu^2+^, which results in quenching of the fluorescence of Ce6 located at 652 nm. However, the addition of arsenate (As(V)) can interact with the oxygen on the carboxyl group outside the ring, leading to the reinstatement of the quenched fluorescence. Consequently, the “turn-off” and “turn-on” of the fluorescence of Ce6 can allow the optional detection of Cu^2+^ and As(V), respectively. In addition, arsenite (As(III)) can interact with the N and O atoms in the Ce6 molecule, causing the Ce6 fluorescence emission peak to be redshifted from 652 nm to 665 nm, so the purpose of detecting As(III) can be accomplished by monitoring Ce6 fluorescence intensity at 665 nm with different As(III) concentrations (Figure 1).

## 2. Results and Discussion

### 2.1. FT–IR

**Figure 2:**
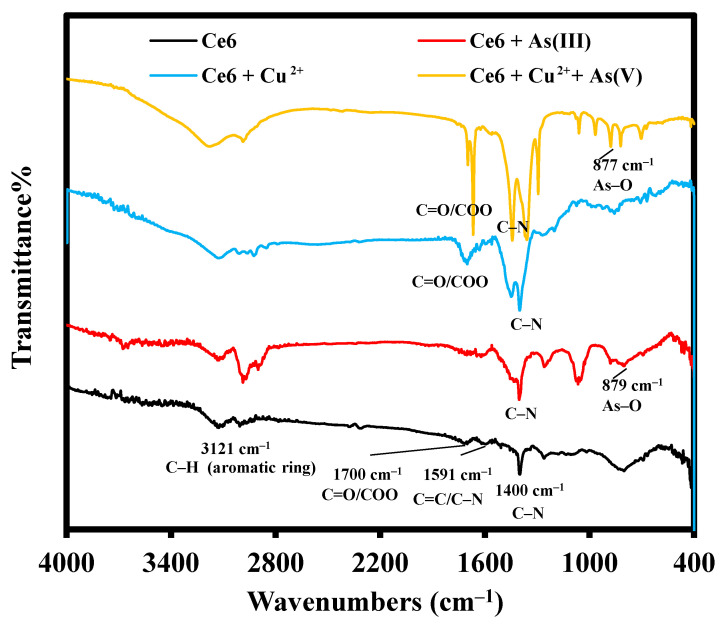
FT–IR spectra of Ce6, Ce6 + As(III), Ce6 + Cu^2+^, and Ce6 + Cu^2+^ + As(V).

The infrared spectra of Ce6, Ce6 + As(III), Ce6 + Cu^2+^, and Ce6 + Cu^2+^ + As(V) are shown in Figure 2. For Ce6, the characteristic peaks at 1400 cm^−1^, 1591 cm^−1^, 1701 cm^−1^, and 2925–3129 cm^−1^ were attributed to the stretching vibration of C–N (aromatic frame), C=C/C=N (aromatic frame), C=O (carboxyl), and C–H (methyl or methylene group) on the aromatic ring [53,54], respectively. The addition of As(III) resulted in a novel vibrational peak at 879 cm^−1^, which is the characteristic stretching vibration peak of As–O [20]. At the same time, the C=N tensile vibration peak at 1591 cm^−1^ disappeared after adding As(III), indicating that the As(III) mainly acted with the carboxyl group outside the ring and the amino group inside the ring to affect the fluorescence characteristics of Ce6. The inclusion of Cu^2+^ in Ce6 brings about the splitting of the C–N characteristic stretching vibration peak at 1400 cm^−1^ and the disappearance of the C=N/C=C characteristic stretching vibration peak at 1591 cm^−1^, indicating that Cu^2+^ mainly interacts with the nitrogen atoms in the porphyrin ring. It is also confirmed by the slight shift (Δδ = 0.08 ppm) of the peak attributed to –NH at 5.37 ppm to the high field after the addition of Cu^2+^ to Ce6 in the ^1^H-NMR spectrum in Figure S1. Upon adding As(V) to the Ce6-Cu^2+^ mixture system, the splitting of the characteristic peaks of C–N (1400 cm^−1^) and –COOH (1701 cm^−1^), along with the recovery of the peaks of –NH (δ 5.37 ppm) and the disappearance of the peaks of –COOH (δ 11.71 ppm) (Figure S1). It is suggested that the As(V) mainly interacts with –COOH outside of the Ce6 ring, which simultaneously affects the coordination of Cu^2+^ with nitrogen atoms in the porphyrin ring.

### 2.2. Feasibility of Fluorescence Detection of As(III), Cu^2+^, and As(V)

**Figure 3:**
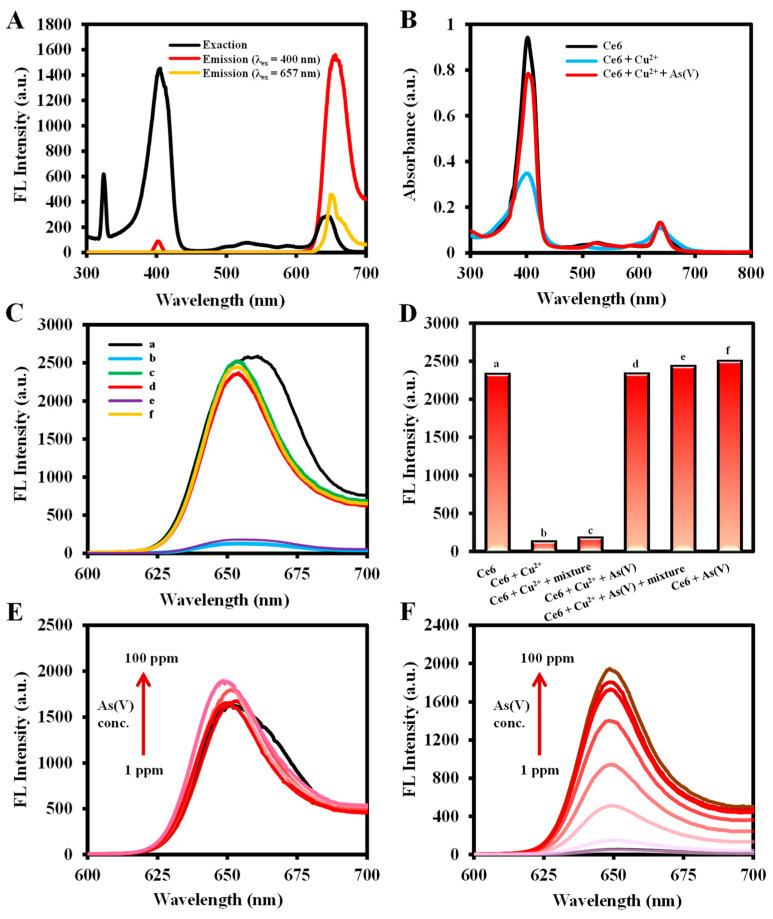
(**A**) Ce6 excitation and emission fluorescence spectroscopy; (**B**) absorbance spectra of Ce6, Ce6 + Cu^2+^, Ce6 + Cu^2+^ + As(V); (**C**) fluorescence spectra and (**D**) histogram of fluorescence intensity at 652 nm (λ_ex_=400 nm) of Ce6 (a), Ce6 + Cu^2+^ (b), Ce6 + As(V) (c), Ce6 + Cu^2+^ + As(V) (d), Ce6 + Cu^2+^ + ion mixture (e), Ce6 + Cu^2+^ + As(V) + ion mixture (f) (ion mixture: Ca^2+^, Mg^2+^, and Mn^2+^, the concentration of all ions was 1 mM in ultrapure water;) (**E**) fluorescence spectra of As(V) + Ce6 and (**F**) fluorescence spectra of As(V) + Ce6 + Cu^2+^. Conditions: the volume of Ce6, Cu^2+^, As(V), and ion mixture are 200 µL, 100 µL, 100 µL, and 100 µL, respectively (if the substance is not present, make up the volume with ultrapure water to 1 mL); Cu^2+^ concentration, 0.1 g/L in ultrapure water for (**B**–**F**); Ce6 concentration, 0.0125 g/L in ethanol for (**B**–**D**), 0.0125 g/L in ethanol for (**A**,**E**,**F**); As(V) concentration, 0.1 g/L in ultrapure water for (**B**–**D**), 0.001g/L to 0.1 g/L in ultrapure water for (**E**,**F**); reaction temperature, 40 °C for (**B**), 50 °C for (**C**,**D**), 30 °C for (**E**,**F**); reaction time, 7 min for (**B**), 5 min for (**C**–**F**); fluorescence spectra were recorded from 600 to 700 nm at an excitation wavelength of 400 nm (excitation/emission slit width: 2.5/10 nm, scan speed: 1200 nm/min, PMT Voltage: 400 V).

As illustrated in Figure 1 and Figure 3A, Ce6 emits an intense red fluorescence at 652 nm under 400 nm excitation wavelength. When Cu^2+^ was added, it bonded with the N atom located in the center of the Ce6 macrocycle [55], weakening the fluorescence emission intensity at 652 nm. As(V) interacts with the carboxylate group, which affects the coordination of Cu^2+^ with the N atoms in the macrocycle, leading to a subsequent recovery of the fluorescence at 652 nm. Figure 3B indicates that the UV absorption at 400 nm was significantly weakened by the addition of Cu^2+^ to Ce6, followed by the recovery of UV absorption with the incorporation of As(V). The fluorescence emission spectra (Figure 3C) showed similar results. The fluorescence intensity of Ce6 (a) was used as a reference, and the fluorescence of Ce6 + Cu^2+^ (b) and Ce6 + Cu^2+^ + ion mixture (Ca^2+^, Mg^2+^, and Mn^2+^) (e) solutions was quenched at 652 nm, from which the ion mixture did not have a significant effect on Cu^2+^ detection. Compared to the fluorescence intensity of the Ce6 + Cu^2+^ (b) system, the Ce6 + Cu^2+^ + As(V) (d) and Ce6 + Cu^2+^ + As(V) + ion mixture (f) solutions recovered their fluorescence at 652 nm, and the ion mixture did not affect the As(V) detection as well (Figure 3D). In addition, Figure 3E,F show the fluorescence emission spectra of different concentrations of As(V) reference standard solution added under the conditions without or with Cu^2+^, indicating that only in the presence of Cu^2+^ does the fluorescence emission intensity change regularly with the concentration of As(V).

As shown in Figure S2A,B, the addition of As(III) causes a redshift in the fluorescence emission wavelength of Ce6, which greatly enhances its fluorescence emission. The As(III) can be detected based on the fluorescence intensity change at 665 nm. However, the addition of As(V) affects the detection of As(III), and this effect cannot be eliminated by introducing the reducing agent ascorbic acid (AA), which limits the detection of As(III) to some degree. In addition, Figure S2C shows that the detection of As(V) is not interfered with by As(III), whereas As(V) affects the detection of As(III) (Figure S2D). Therefore, this method, based on Ce6-Cu^2+^, is better for selective detection of As(V).

### 2.3. Optimization of Detection Condition

**Figure 4:**
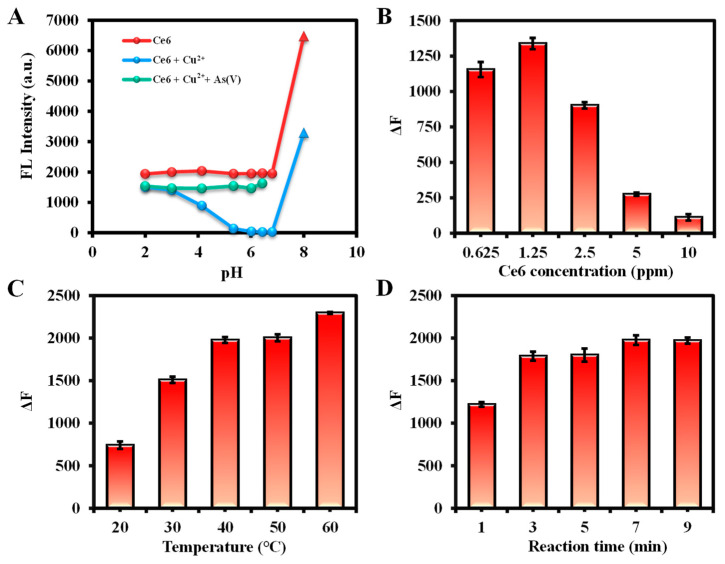
(**A**) The pH dependence study of Ce6, Ce6 + Cu^2+^, and Ce6 + Cu^2+^ + As(V), where circles indicate the fluorescence intensity at 652 nm and triangles at 665 nm. Conditions: the volume of Ce6, ultrapure water, Cu^2+^, H^+^/OH^−^ and As(V) are 200 µL, 500 µL, 100 µL,100 µL, and 100 µL, respectively; Ce6 concentration, 3.125 ppm in ethanol; Cu^2+^ concentration, 100 ppm in ultrapure water; As(V) concentration, 10 ppm in ultrapure water; reaction temperature, 30 °C; reaction time, 5 min, (**B**) The effects of Ce6 concentration, (**C**) reaction temperature, and (**D**) reaction time on the fluorescence intensity for Cu^2+^ detection. Conditions: the volume of Ce6, ultrapure water, and Cu^2+^ are 200 µL, 700 µL, and 100 µL, respectively; Ce6 concentration, 6.25 ppm in ethanol for (**C**,**D**); Cu^2+^ concentration, 10 ppm in ultrapure water for (**B**–**D**); reaction temperature, 30 °C for B, 40 °C for (**C**); reaction time, 5 min for (**B**,**C**). Fluorescence spectra were recorded from 600 to 700 nm at an excitation wavelength of 400 nm (excitation/emission slit width: 2.5/10 nm, scan speed: 1200 nm/min, PMT Voltage: 400 V).

The variables involved in the detection were systematically examined to improve the accuracy and sensitivity of the assay. The effects of pH on three systems—Ce6, Ce6 + Cu^2+^, and Ce6 + Cu^2+^ + As(V)—were first explored, and the results are summarized in Figure 4A. The fluorescence intensity of Ce6 and Ce6 + Cu^2+^ + As(V) was stable at pH ≤ 7, but the fluorescence emission peaks of Ce6 itself were redshifted and greatly increased its fluorescence emission intensities at pH > 8, which might be due to the deprotonation under alkaline conditions [38]. In addition, Cu^2+^ has a significant quenching effect on Ce6 when the pH is from 5 to 7, which is more stable for the whole detection system. In summary, the fluorescence intensity is stable at pH = 5–7 for the Ce6, Ce6+Cu^2+^, and Ce6 + Cu^2+^ + As(V) detection systems, so the ultrapure water (pH = 6.80) was used as the dilution solution in this study.

For the Cu^2+^ detection system, the effects of Ce6 concentration, reaction temperature, and reaction time were investigated. Optimal conditions were chosen based on the fluorescence intensity change value (ΔF = F_0_ − F) at 652 nm with and without Cu^2+^ (F and F_0_) in Ce6 solution. Aggregation of Ce6 at high concentrations may lead to the weakening of the fluorescence emission intensity [56]. As shown in Figure 4B, the ΔF value reached a maximum at a Ce6 concentration of 1.25 ppm. As the reaction temperature and time increased, ΔF also gradually enhanced and reached equilibrium at 40 °C and 7 min, respectively (Figure 4C,D). Therefore, the reaction conditions for Cu^2+^ detection were optimized with a dosage of Ce6 at 1.25 ppm, a reaction temperature of 40 °C, and react for 7 min. Similarly, based on the variation of fluorescence intensity at 665 nm in the existence or absence of As(III), the optimized conditions for determination of As(III) were as follows: 2.5 ppm of Ce6, 30 °C of reaction temperature, and 1 min of reaction time (Figure S3). 

**Figure 5:**
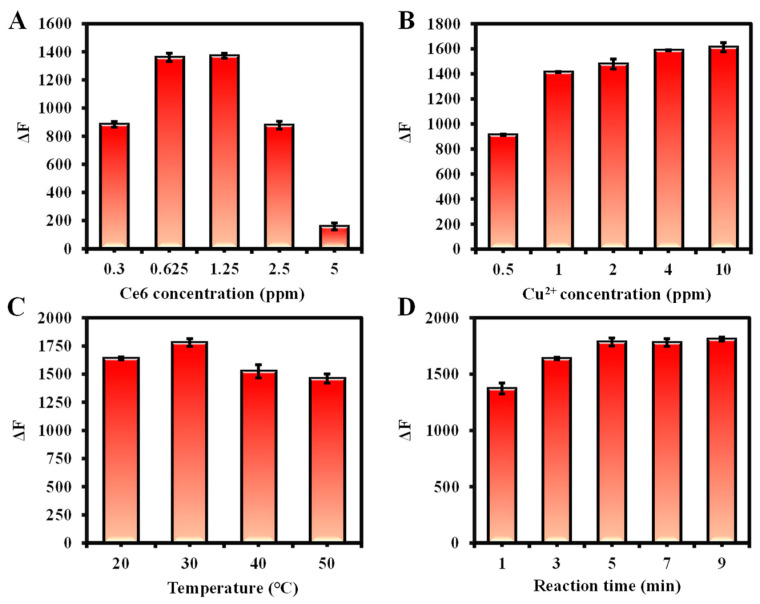
(**A**) The effects of Ce6 concentration, (**B**) Cu^2+^ concentration, (**C**) reaction temperature, and (**D**) reaction time on the fluorescence intensity for As(V) detection. Conditions: the volume of Ce6, ultrapure water, Cu^2+^, and As(V) are 200 µL, 600 µL, 100 µL, and 100 µL, respectively; Ce6 concentration, 3.125 ppm in ethanol for (**B**–**D**); Cu^2+^ concentration, 100 ppm in ultrapure water for (**A**,**C**,**D**); As(V) concentration, 100 ppm in ultrapure water for (**A**–**D**); reaction temperature, 30 °C for (**A**,**B**,**D**); reaction time, 7 min for (**A**–**C**); fluorescence spectra were recorded from 600 to 700 nm at an excitation wavelength of 400 nm (excitation/emission slit width: 2.5/10 nm, scan speed: 1200 nm/min, PMT Voltage: 400 V).

Furthermore, the conditions for As(V) detection were optimized by observing the change in fluorescence at 652 nm (ΔF = F − F_0_) with or without the addition of As(V) (F and F_0_) in the presence of a certain concentration of Cu^2+^. First, the effect of Ce6 concentration on the detection was investigated in the presence of 10 ppm Cu^2+^, and it was found that the recovery of fluorescence intensity by As(V) was the strongest at 0.625 ppm, so 0.625 ppm was considered as the Ce6 dosage. Subsequently, the 10 ppm of Cu^2+^ was chosen due to its almost complete quenching of Ce6 fluorescence intensity. Finally, ΔF gradually enhanced with the increase in temperature and stabilized at 40 °C. Similarly, the reaction time can be selected as 7 min. Consequently, 0.625 ppm of Ce6, 10 ppm of Cu^2+^, reaction temperature of 30 °C, and reaction time of 5 min were used as subsequent detection conditions for As(V) (Figure 5).

### 2.4. Sensitivity of Ce6 for As(III), Cu^2+^, and As(V) Detection

**Figure 6:**
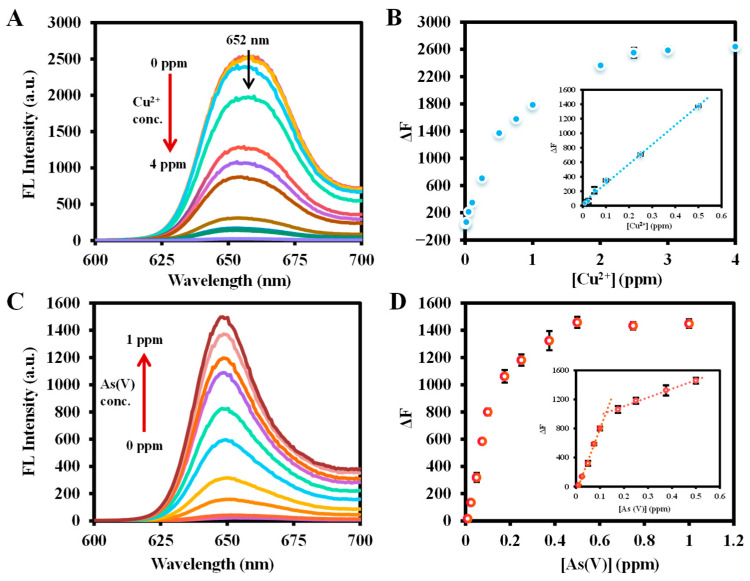
(**A**) Fluorescence emission spectrum of Ce6 at different Cu^2+^ concentrations and (**B**) linear plot of ΔF of Cu^2+^ concentration. Conditions: the volume of Ce6, ultrapure water, and Cu^2+^ are 200 µL, 700 µL, and 100 µL, respectively; Ce6 concentration, 6.25 ppm in ethanol for (**A**,**B**); Cu^2+^ concentration, 0 ppm to 4 ppm in ultrapure water for A and B; reaction temperature, 40 °C for (**A**,**B**); reaction time, 7 min for (**A**,**B**). (**C**) The fluorescence emission spectrum of Ce6 + Cu^2+^ with different As(V) concentrations and (**D**) the linear plot of ΔF of As(V) concentration. Conditions: the volume of Ce6, ultrapure water, Cu^2+^, and As(V) are 200 µL, 600 µL, 100 µL, and 100 µL, respectively; Ce6 concentration, 3.125 ppm in ethanol for (**C**,**D**); Cu^2+^ concentration, 100 ppm in ultrapure water for (**C**,**D**); As(V) concentration, 0 ppm to 1 ppm in ultrapure water for (**C**,**D**); reaction temperature, 30 °C for (**C**,**D**); reaction time, 5 min for (**C**,**D**). Fluorescence spectra were recorded from 600 to 700 nm at an excitation wavelength of 400 nm (excitation/emission slit width: 2.5/10 nm, scan speed: 1200 nm/min, PMT Voltage: 400 V).

Cu^2+^ plays a vital role in different metabolic processes in living organisms, but high concentrations of Cu^2+^ entering the human body can have serious toxic effects [25]. The intensity of the red fluorescence emission of Ce6 at 652 nm gradually decreased with the continuous addition of Cu^2+^ (Figure 6A). The fluorescence emission intensities without and with the addition of Cu^2+^ were set as F_0_ and F. The concentration of Cu^2+^ was used as the horizontal coordinate, and the fluorescence change value ΔF = F_0_ − F after the inclusion of Cu^2+^ was plotted as the vertical coordinate in a scatter plot (Figure 6B). It is observed that a good linear correlation with ΔF was presented in the concentration range of Cu^2+^ of 0.04–2 μM (0.01–0.5 ppm), yielding a linear equation of Y = 2680.1 X + 38.841 (R^2^ = 0.9952). The limit of detection (LOD) was assessed to be 0.212 μM (53.1 ppb) using the 3*σ*/*S* (*S* is the calibration curve slope, and *σ* is the standard deviation of the zero level), which was much lower than that of 20 μM specified by the United State Environmental Protection Agency (EPA) [24].

With the addition of As(V), the fluorescence emission intensity at 652 nm quenched by the Ce6-Cu^2+^ system was gradually restored (Figure 6C). Based on the optimal conditions for detecting As(V), the fluorescence intensity of Cu^2+^ (10 ppm) quenching was set to F_0_, and the intensity of fluorescence emission after the addition of As(V) was set to F. A scatter plot was made with the concentration of As(V) as the abscissa and the change in fluorescence intensity after adding As(V) (ΔF = F − F_0_) as the vertical coordinate. As shown in Figure 6D, the concentration range of As(V) from 0.01 to 0.175 ppm and 0.25 to 0.5 ppm showed a good linear relationship with ΔF with a linear equation of Y = 8785.1 X − 85.91 (R^2^ = 0.9962) and Y = 1201.9 X − 865.96 (R^2^ = 0.9937), respectively, and the LOD (S/N = 3) was calculated to be 1.375 ppb, which was below the WHO regulation for arsenic in water (10 ppb) and better than other methods of detecting arsenic using small-molecular ligands (Table 1).

The fluorescence emission peak of Ce6 was redshifted from 652 nm to 665 nm by As(III), so the changing fluorescence intensity at 665 nm in the presence or absence of As(III) was used to construct the scatter plot. The intensity of fluorescence at 665 nm gradually increased with the addition of As(III) and was linear in the range of As(III) concentration from 0.01 to 2.5 ppm with the fitting equation Y = 755.14X + 7.9028 (R^2^ = 0.9962), and a LOD of 0.089 ppm was obtained (Figure S3). It can be seen that the sensitivity of this method for As(III) is not excellent, so the detection mechanism was not explored further in this paper.

### 2.5. The Binding Study of Ce6 and Cu^2+^/As(V)

**Figure 7:**
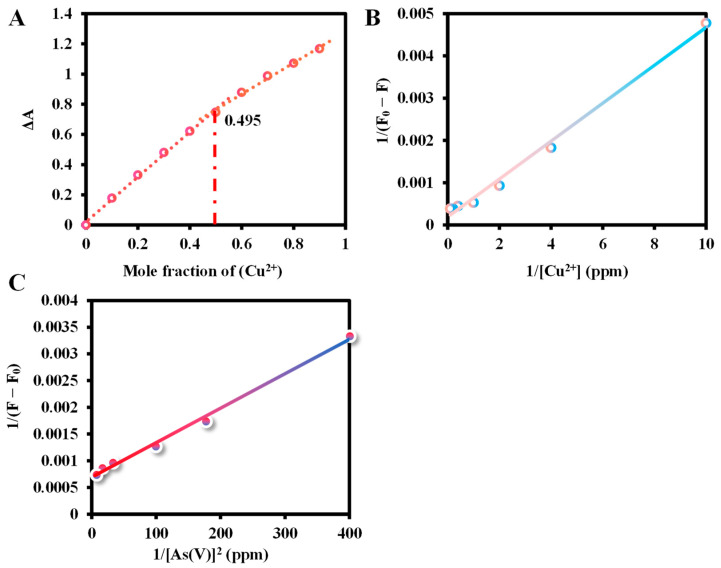
(**A**) Job’s plot data for evaluating the stoichiometry of Ce6-Cu^2+^. The total concentration of Ce6 and Cu^2+^ was 10 μM. The wavelength of absorbance is 400 nm. (**B**) B–H plot of the complex of Ce6-Cu^2+^. Conditions: the concentration of Ce6 was 1.25 ppm. The reaction time was 7 min at 40 °C, and the fluorescence spectrum of 600–700 nm was recorded under 400 nm excitation. (**C**) B–H plot of the complex between Ce6-Cu^2+^ and As(V). Conditions: the concentrations of Ce6 and Cu^2+^ were 0.625 ppm and 10 ppm, respectively. The reaction time was 5 min at 30 °C, and the fluorescence spectrum of 600–700 nm was recorded under 400 nm excitation. (excitation/emission slit width: 2.5/10 nm, scan speed: 1200 nm/min, PMT Voltage: 400 V).

The molar fractions of Ce6 and Cu^2+^ were gradually varied in the absorption spectra of the equimolar solution (10 μM) to observe the complexation of Ce6 with Cu^2+^. As shown in Job’s plot of Figure 7A, the molar fraction value of copper ions is 0.495, which is in accordance with the stoichiometric ratio of 1:1, indicating that the Cu ions are monocoordinated with the N atoms on the central cavity of the Ce6 macrocycle. Moreover, the Bensi-Hildebrand (B–H) equation can be used to calculate the binding ratio and the binding constant, which are expressed as 1/(F − F_0_) = 1/(F_max_ − F_0_) + 1/*K*[I^−^]^n^(F_max_ − F_0_), where F_0_, F_max_, and F are the fluorescence emission intensities in the absence, saturated Cu^2+^/As(V) is present and a given amount of Cu^2+^/As(V) concentration is added, respectively, *K* is the binding constant, the [I^−^] is the Cu^2+^/As(V) concentration, n is the binding ratio [38]. The B–H plot of the linear relationship between Cu^2+^ concentration and 1/(F_0_ − F) confirms the 1:1 binding between Ce6 and copper, and the binding constant was obtained to be 5 × 10^2^ (g/L)^−1^ (1.248 × 10^5^ M^−1^) from the B–H equation (Figure 7B). As shown in Figure 7C, a B–H analysis based on the square of the As(V) concentration yields a well-fitted curve, indicating a binding ratio of 1:2 between Ce6-Cu^2+^ and As(V) [57]. The apparent binding constant for As(V) was estimated to be 1.167 × 10^8^ (g/L)^−2^ (2.35 × 10^12^ M^−2^).

### 2.6. Detection Mechanisms

**Figure 8:**
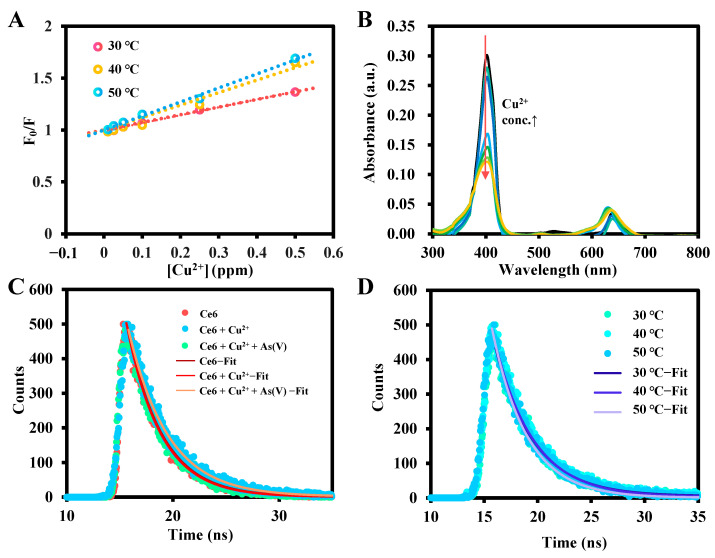
(**A**) Stern–Volmer diagrams of Cu^2+^ at different temperatures. (**B**) UV–vis absorption spectra of the Ce6 in the presence of different Cu^2+^ concentrations. (**C**) Fluorescence decay curves of Ce6, Ce6 + Cu^2+^, and Ce6 + Cu^2+^ + As(V) and (**D**) Fluorescence decay curves of Ce6 + Cu^2+^ of different temperatures.

The detection mechanism of Cu^2+^ based on Ce6 was investigated. It can be seen from the gradual decrease in fluorescence intensity with increasing Cu^2+^ concentration through dynamic or static quenching. To further explore the quenching mechanism, the Stern–Volmer equation was introduced: F_0_/F = 1 + *K_q_*τ_0_[Q] = 1 + *K_sv_*[Q], where F and F_0_ are the intensity of fluorescence with or without the quenching agent, [Q] is the concentration of Cu^2+^, *K_q_* is the quenching constant, τ_0_ is the average lifetime of Ce6, and *K_sv_* is the Stern–Volmer constant. Based on the Stern–Volmer curves of Cu^2+^ at different temperatures in Figure 8A, the *K_q_* values were calculated to be 2.222 × 10^11^ (30 °C), 3.639 × 10^11^ (40 °C), and 4.095 × 10^11^ (50 °C), respectively, i.e., the quenching constants increased gradually with increasing temperature, which is in accordance with the characteristics of collisional quenching [58]. To further investigate the quenching mechanism, the UV absorption spectra of Ce6 in the presence of different concentrations of Cu^2+^ were recorded (Figure 8B), and it was found that the addition of Cu^2+^ brought about a gradual decrease of the absorption spectrum without significant redshift and blueshift, which ruled out the probability of the formation of a basal complex between Ce6 and Cu^2+^ [59].

Dynamic quenching of any non-radiative process in the quencher interacts with the excited state of the fluorophore, resulting in changes in steady-state fluorescence intensity and fluorescence lifetime. Static quenching, on the other hand, is that inhibits the formation of the excited state of a fluorophore, therefore completely quenching its original fluorescence, with the formation of a stable complex between the quencher and the fluorophore and no change in fluorescence lifetime [60]. The results depicted in Figure 8C show that the fluorescence lifetime of Ce6 maintains a mono-exponential variation. After the introduction of Cu^2+^, the fluorescence lifetime changed from 3.26 ns to 3.72 ns, and its lifetime did not change with temperature change (Figure 8D), which is consistent with dynamic quenching. In addition, this enhanced fluorescence lifetime may be due to the slow internal electron transfer caused by the coordination of Cu^2+^ with the N atom in the center of the Ce6 macrocycle [61], which may also be responsible for the fluorescence quenching. Interestingly, fluorescence recovery occurs with the addition of As(V) to the Ce6-Cu^2+^ system, and its fluorescence lifetime returns to a level comparable to that of Ce6, which may be owing to the interaction of As(V) with the carboxylate group outside the ring improves the planarity and rigidity of the complex [62], leading to an increase in the rate of electron transfer, and resulting in the “turn-on” fluorescence.

### 2.7. Interference and Selectivity Study

To evaluate the selectivity of the reaction system for the detection of Cu^2+^ by Ce6, the fluorescence response of interfering substances, including metal and non-metal ions and nucleotides present in real samples, to Ce6 was assessed. The concentrations of metal ions and nucleotides (10 ppm) are comparable to those of Cu^2+^ (10 ppm). As shown in Figure S4, metal ions such as Cd^2+^, Mn^2+^, Mg^2+^, Co^2+^, Ni^2+^, Zn^2+^, K^+^, Na^+^, and Ca^2+^ do not produce significant quenching of the fluorescence intensity of Ce6, which shows excellent selectivity for Cu^2+^. Furthermore, except for As(V) and adenine, other metal ions interfered slightly with the detection of the Cu^2+^ system. Among them, adenine mainly interferes with Cu^2+^ through coordination, leading to a decrease in the concentration of Cu^2+^ in the solution [63], whereas the addition of As(V) produces a strong recovery of the fluorescence intensity.

**Figure 9:**
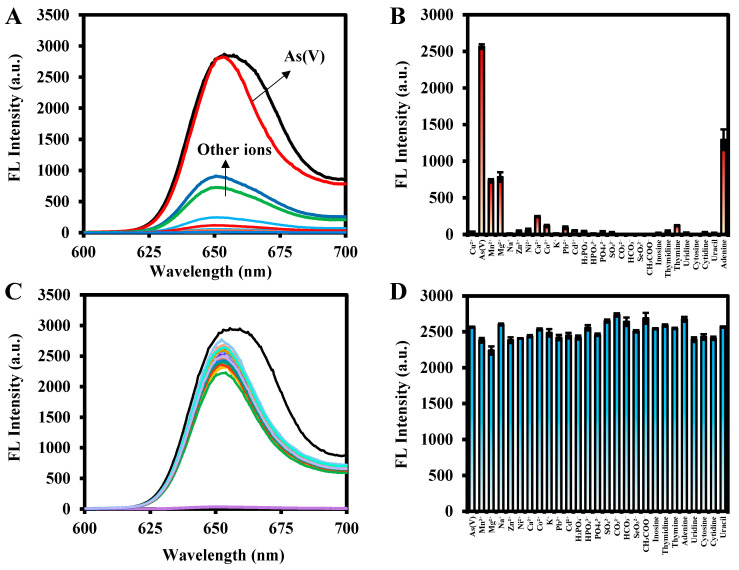
(**A**,**B**) Selectivity and (**C**,**D**) interference assays of Ce6-Cu^2+^ to 10 ppm As(V) and other different anions (10 ppm).

For the detection of As(V), the selectivity and interferences of metal ions, acid ions, and nucleotides in the detection system were investigated, respectively. The concentrations of metal ions, acid ions, and nucleotides (10 ppm) were comparable to those of As(V) (10 ppm). As shown in Figure 9A,B, the presence of As(V) significantly enhanced the fluorescence intensity of the Ce6-Cu^2+^ complex at 652 nm, except for a slight enhancement of fluorescence at 652 nm by Ca^2+^, Mg^2+^, Mn^2+^, and adenine (with which Cu^2+^ is coordinated). In addition, other metal ions such as Co^2+^, Cd^2+^, Mn^2+^, and Mg^2+^, and acid radical ions such as H_2_PO_4_^−^, HPO_4_^2−^, PO_4_^3−^, CO_3_^2−^, and SeO_3_^2−^, and nucleotides such as inosine, thymidine, and uridine had negligible effect on the fluorescence intensity of Ce6-Cu^2+^ complex, which indicated that the method exhibited an excellent selectivity for As(V). Furthermore, the addition of other interfering substances to the detection system of As(V) and the variation of maximum fluorescence emission intensity was plotted. It was noted that the presence of other metal ions, acid ions, and nucleotides had no significant effect on the As(V) detection (Figure 9C,D).

Finally, the interference and selectivity study for As(III) detection was also performed using Ce6 and a certain concentration (10 ppm) of interfering elements (Figure S5). The selectivity studies revealed that Ce6 is highly selective for As(III) except for phosphate and hydrogen phosphate. However, interference studies have shown that the assay is susceptible to interference from most ions. This implies that direct detection of As(III) using Ce6 has some limitations.

### 2.8. Real Sample Analysis

For the evaluation of the practical application, the assay was used to analyze actual water and soil samples to which known amounts of reference standard solutions of As(III), Cu^2+^, and As(V) were added (Table 2, Tables S1 and S2), respectively. The determination was calculated by evaluating their recoveries and relative standard deviations (RSD). The recovery of As(III) added was acquired from 82.2% to 118.5%, whereas for Cu^2+^, it was from 89.0% to 110.4%, and As(V) was from 80.2% to 116.3% at different known amounts, and all of them had the RSDs less than 7.6%, which demonstrated that the application of this method in real samples analysis was quite feasible.

## 3. Materials and Methods

### 3.1. Chemicals and Instruments

The details of chemicals and instruments used in this study are presented in the Supplementary Material.

### 3.2. Fluorescence Detection of As(III) with Ce6

The detection of As(III) was based on the variation of fluorescence intensity at 665 nm. Before the test, 200 μL of Ce6 solution (12.5 mg/L) was added to a 1.5-mL centrifuge tube, followed by 700 μL of water and 100 μL of As(III) reference standard solution, and incubated for 1 min at 30 °C in a water bath. Finally, the fluorescence emission spectra were recorded at 600–700 nm. At the same time, measuring the fluorescence intensity of the corresponding NaHCO_3_ (matrix of the As(III) reference standard solution) and subtracting it according to the same assay procedure gives the fluorescence intensity for detecting As(III). (λ_ex_: 400 nm; excitation/emission slit width: 2.5/10 nm, scan speed: 1200 nm/min, PMT Voltage: 400 V).

### 3.3. Fluorescence Detection of Cu^2+^ with Ce6

The detection of Cu^2+^ was based on the difference in fluorescence quenching intensities of Ce6 in the presence of different Cu^2+^ concentrations in the solution. In a 1.5-mL centrifuge tube, 200 μL of Ce6 (6.25 mg/L) ethanol solution, 700 μL of ultrapure water, and 100 μL of Cu^2+^ reference standard solution was added successively and placed in a water bath at 40 °C for 7 min. Then, the fluorescence emission spectra of 600–700 nm were recorded.

### 3.4. Fluorescence Detection of As(V) with Ce6-Cu^2+^

The determination of As(V) was based on the different recovered fluorescence intensities of the Ce6-Cu^2+^ system in the existence of different As(V) concentrations in solution. A total of 200 μL of Ce6 (3.125 mg/L) ethanol solution, 600 μL of ultrapure water, 100 μL of Cu^2+^ (0.1 g/L), and 100 μL of As(V) reference standard solution were added into a 1.5-mL centrifuge tube in a 30 °C water bath for 5 min. Then, the fluorescence emission spectra were recorded at 600–700 nm.

### 3.5. Interference Analysis

Interfering substances that may be present in actual samples, including metal ions (Mn^2+^, Mg^2+^, Na^+^, Zn^2+^, Ni^2+^, Ca^2+^, Co^2+^, K^+^, Pb^2+^, and Cd^2+^), non-metal ions (H_2_PO_4_^−^, HPO_4_^2−^, PO_4_^3−^, SO_4_^2−^, CO_3_^2−^, HCO_3_^−^, SeO_3_^2−^, and CH_3_COO^−^), and amino acids (inosine, thymidine, thymine, adenine, uridine, cytosine, cytidine, and uracil) were selected to determine the selectivity and specificity of the method according to the assay procedure described above. The concentrations of all interfering elements were 10 ppm.

### 3.6. Detection of Cu^2+^, As(V), and As(III) in Real Sample

To verify the practicability of the developed method for the analysis of real samples, water from Yun Lake and Jin Lake at Chongqing University, as well as the surrounding soil, were collected for measurement. Before the test, the water samples were passed through a 0.22 μm filter membrane twice for spare. The soil samples were dried at 90 °C for 2 h and then ground into powder. Disperse 500 mg of dried soil powder in 9 mL of ultrapure water and ultrasonicate for 10 min. The supernatant was passed through a 0.22 μm filter membrane three times after 30 min of standing.

Different As(III) reference standard solutions (1, 5, and 25 ppm), Cu^2+^ solutions (0.5, 1.0, and 2.5 ppm), and As(V) reference standard solutions (0.25, 0.5, and 0.75 ppm) were added to the pre-treated real samples and analyzed using the same assay procedure described above. The reliability of the established analytical method can be estimated by the RSD, and the sample spiked recoveries.

## 4. Conclusions

In this paper, the chlorophyll degradation product Ce6 was successfully introduced for the first time for fluorescence “turn-off” and “turn-on” detection of Cu^2+^ and arsenic. The detection limit of Cu^2+^ based on fluorescence quenching was 0.212 μM. The stoichiometric ratio of Cu^2+^ to Ce6 was calculated to be 1:1 by Job’s curve and B–H equation, and the binding constant was 1.248 × 10^5^ M^−1^. The mechanism of Cu^2+^ quenching of Ce6 fluorescence was analyzed using fluorescence lifetimes and the Stern–Volmer equation to be a dynamic quenching due to photoinduced electron transfer. On the other hand, As(V) restored the fluorescence of Ce6-Cu^2+^ with the LOD of 1.375 ppb and a binding ratio of 1:2 between Ce6-Cu^2+^ and As(V) and a binding constant of 2.35 × 10^12^ M^−2^ calculated by the B–H equation. The mechanism of fluorescence restoration was attributed to the improvement of rigidity and planarity in the molecular structure. The developed method has excellent selectivity and sensitivity, which can be used for the determination of Cu^2+^ and arsenic in water and soil samples.

## Figures and Tables

**Table 1:** Detection of arsenic by fluorescent probes based on various small-molecule receptors.

Receptors	Detection Ion	Liner Range (ppm)	LOD (ppb)	Ref.
Acf and RhB	As(V)	0.04–0.09	10	[3]
APSAL	As(V)	4.65–37.2	557.73	[5]
AF1	As(III)	– ^a^	0.24	[9]
SB	As(III)	0.1–14	3.12	[33]
DMBD	As(III)	0–500	0.22	[32]
HL	As(III)	0–015	4.1	[35]
2′,7′-dichlorofluorescein	As(III)	0.005–0.05	0.102	[37]
Ce6 Ce6-Cu^2+^	As(III) As(V)	0.01–2.5 0.01–0.25	89 1.375	This work

Acf: Acriflavine, RhB: Rhodamine B; APSAL: (4E)-4-(2-hydroxybenzylideneamino)-1,2-dihydro-2,3-dimethyl-1-phenylpyrazol-5-one; AF1: 7-(diethylamino)-3-(5-(trifluoromethyl)-2,3-dihydrobenzothiazol-2-yl)-2H-chromen-2-one; SB: anthranilic acid-based Schiff base; DMBD: 3′,6′-bis(diethylamino)-2-{[(1E)-(4,5-dimethyl-2-furyl)methylene]amino}spiro[isoindole-1,9′-xanthen]-3(2H)-one; HL: diformyl-p-cresol-based receptor. ^a^ not mentioned in the reference.

**Table 2:** Determination of As(V) in real water and soil samples.

Samples	Added (ppm)	Founded (ppm)	Recovery ^a^ (%)	RSD (%) (*n* = 3)
Yun Lake water	0.00	– ^b^		
	0.01	0.0091	91.3	4.1
	0.05	0.0513	102.6	7.6
	0.10	0.0961	96.1	4.9
Jin Lake water	0.00	– ^b^		
	0.01	0.0119	118.9	2.7
	0.05	0.0400	80.1	5.6
	0.10	0.0815	81.5	1.8
Yun Lake Soil	0.00	– ^b^		
	0.01	0.0106	106.3	2.7
	0.05	0.0454	90.9	3.2
	0.10	0.0897	89.7	2.1
Jin Lake Soil	0.00	– ^b^		
	0.01	0.0111	111.4	5.7
	0.05	0.0420	84.1	6.6
	0.10	0.1049	104.9	6.4

^a^ Recovery = (founded concentration-original concentration)/added concentration × 100%. ^b^ The detection value is below the quantification limit of this method.

## Data Availability

The data presented in this study are contained within the article.

## Supplementary Materials

The following supporting information can be downloaded at https://www.mdpi.com/article/10.3390/molecules29051015/s1. Table S1. Detection of Cu^2+^ by fluorescence based on various small-molecule receptors; Table S2. Determination of Cu^2+^ in real water and soil samples; Table S3. Determination of As(III) in real water and soil samples; Figure S1. The ^1^H-NMR spectra of Ce6 (a), Ce6 + Cu^2+^ (b), and Ce6 + Cu^2+^ + As(V) (c). The inset shows the structure of the Ce6 molecule; Figure S2. (A) The effect of different substances on the fluorescence spectrograms of Ce6 and (B) the corresponding histograms; (C) Influence of As(III) on the detection of As(V); (D) Influence of As(V) on the detection of As(III); Figure S3. The effects of (A) Ce6 concentration, (B) reaction temperature, and (C) time on the fluorescence intensity for As(III) detection. (D) Fluorescence emission titration of Ce6 with different concentrations of As(III) at an excitation wavelength of 400 nm in ultrapure water and (E and F) linear plot of ΔF (at 665 nm) of As(III) concentration; Figure S4. (A and B) Selectivity and (C and D) interference assays of Ce6 to 10 ppm of Cu^2+^ and other different anions; Figure S5. (A and B) Selectivity and (C and D) interference assays of Ce6 to 10 ppm of As(III) and other different anions. References [64,65,66] are cited in the Supplementary Materials.
