# Supplementary material for: Detection of Arsenic(V) by Fluorescence Sensing Based on Chlorin e6-Copper Ion
Source: Molecules. 2024 Feb 26;29(5):1015. doi: 10.3390/molecules29051015 (PMC10934122; doi:10.3390/molecules29051015)
Supplement: Supplementary file 1 [file molecules-29-01015-s001.zip › molecules-2873864-supplementary.pdf]

Supplementary Material

Detection of arsenic(V) by fluorescence sensing based on Chlorin e6-Copper ion

Mao-Ling Luo [1], Guo-Ying Chen [1], Jia-Li Wang [1], Tong-Qing Chai [1], Zheng-Ming Qian [2], Wen-Jia Li [2,*], Feng-Qing Yang [1,*]

[1] School of Chemistry and Chemical Engineering, Chongqing University, Chongqing 401331, China; 20185486@cqu.edu.cn (M.-L.L.); 20221801017@stu.cqu.edu.cn (G.-Y.C.); 202118021010@cqu.edu.cn (J.-L.W.); 20175531@cqu.edu.cn (T.-Q.C.); qianzhengming@hec.cn (Z.-M.Q.)

[2] Dongguan HEC Cordyceps R&D Co., Ltd, Dongguan, Guangdong 523850, China

* Correspondence: fengqingyang@cqu.edu.cn (F.-Q.Y.); liwenjia@hec.cn (W.–J. L.)

1. Materials and Methods

1.1 Chemicals and materials

Chlorin e6 ($C_{34}H_{36}N_4O_6$, Ce6) was obtained from Shanghai D&B Biological Science and Technology Co., Ltd. (Shanghai, China). Arsenate (V) (H_3AsO_4, 1001 μg/mL) and arsenic(III) reference standard solution (H_3AsO_3, 1000 μg/mL) was purchased from Beijing North Weiye Metrology Technology Research Institute Co., Ltd. (Being, China). Copper (II) sulfate pentahydrate ($CuSO_4{\cdot}5H_2O$) was purchased from Chongqing Chuandong Chemical (Group) Co., Ltd. (Chongqing, China). Calcium chloride ($CaCl_2$, ≥ 96%) was obtained from Tianjin Damao Chemical Reagent Factory (Tianjin, China). Zinc acetate dihydrate ($CH_3COOZn{\cdot}2H_2O$) was purchased from Shanghai Titan Scientific Co., Ltd. (Shanghai, China). Sodium selenite (Na_2SeO_3, ≥ 98%) was purchased from Shanghai Meryer Biochemical Technology Co., Ltd. (Shanghai, China). Sodium hydroxide (NaOH, ≥ 98%), potassium dihydrogen phosphate (KH_2PO_4, ≥ 99.5%), disodium hydrogen phosphate (Na_2HPO_4, ≥ 99%), trisodium phosphate (Na_3PO_4, ≥ 98%), potassium chloride (KCl, ≥ 99.5%), sodium chloride (NaCl, ≥ 99.5%), disodium edetate dihydrate ($C_{10}H_{14}N_2O_8Na_2{\cdot}2H_2O$, ≥ 99%), sodium carbonate (Na_2CO_3, ≥ 99.8%), sodium bicarbonate ($NaHCO_3$, ≥ 99.5%), sodium sulfate (Na_2SO_4, ≥ 99%), magnesium sulfate heptahydrate ($MgSO_4{\cdot}7H_2O$, ≥ 99%)), manganese sulfate ($MnSO_4{\cdot}H_2O$, ≥ 99%), nitric acid (HNO_3, 65.0%~68.0%), and hydrochloric acid (HCl, 36.0%–38.0%) were obtained from Chengdu Chron Chemical Co., Ltd. (Chengdu, China). Nickel chloride hexahydrate ($NiCl_2{\cdot}6H_2O$, 99%) and cadmium chloride ($CdCl_2{\cdot}5H_2O$, 98%) were obtained from Shanghai Macklin Biochemical Technology Co., Ltd. (Shanghai, China). Adenine phosphate (VB4, 98%) was obtained from Shanghai Yuanye Biotechnology Co., Ltd. (Shanghai, China). Sodium acetate (CH_3COONa, 99.5%), and cobalt nitrate hexahydrate ($Co(NO_3)_2{\cdot}6H_2O$, 99.99%) were obtained from Shanghai Aladdin Biochemical Technology Co., Ltd.

(Shanghai, China). Thymidine (99%), cytidine (99%), uridine (99%), uracil (99%), cytosine (99%), thymine (99%), inosine (99%), and adenine (99%) were obtained from Sinopharm Chemical Reagent Co., Ltd. (Shanghai, China). L-cysteine (L-Cys, 99.0%) was obtained from Tianjin Guangfu Fine Chemical Research Institute (Tianjin, China). All substances were prepared using ultrapure water except Ce6, which was dissolved in anhydrous ethanol. The pH of this experiment was adjusted by NaOH (1 M) and HNO_3 (1.5 M) solutions.

1.2 Instruments

The optical characteristics of Ce6 were investigated on a UV-5500 PC spectrophotometer (Shanghai Metash Instruments Co., Ltd., Shanghai, China) and an F-7100 fluorescence spectrophotometer (Hitachi Ltd., Tokyo, Japan). A DHG-9146A electric heating constant temperature blast drying shaker (Shanghai Longyue Instrument Equipment Co., Ltd., Shanghai, China) and a smart thermostatic water bath (Chang-sha Mitr Instrument Equipment Co., Ltd., Changsha, China) were used for tempera-ture control. The ultrasonic cleaner used in this study was obtained from Jiangsu Kunshan Jielimei Ultrasonic Instrument Co., Ltd. (Kunshan, China). The ultrapure water prepared through a water purification system (ATSelem 1820A, Antesheng Environmental Protection Equipment, Chongqing, China) was used for all the experiments. Fourier transform infrared (FT-IR) spectroscopy analysis was conducted on a Nicolet iS10 spectrometer (Shimadzu, Japan).

2. Supplementary figures

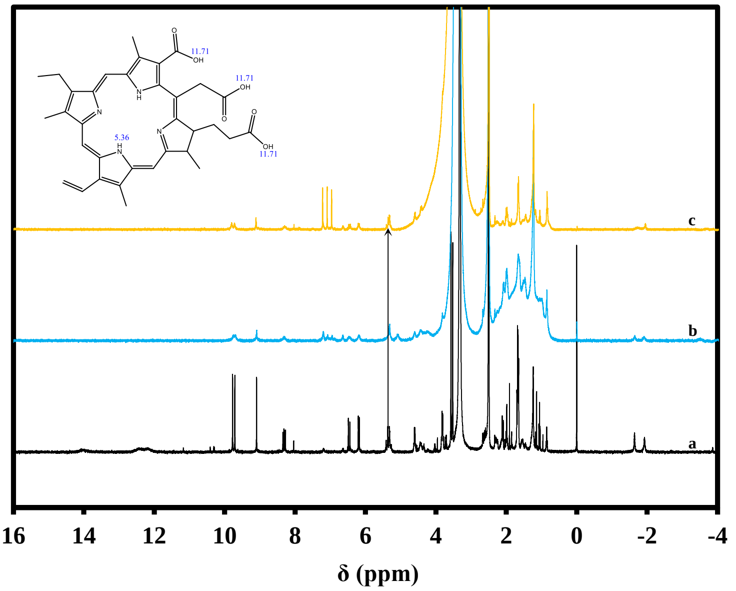

Figure S1. The ^{1}H-NMR spectra of Ce6 (a), Ce6 + Cu^{2+} (b), and Ce6 + Cu^{2+} + As(V) (c). The inset shows the structure of Ce6 molecule.

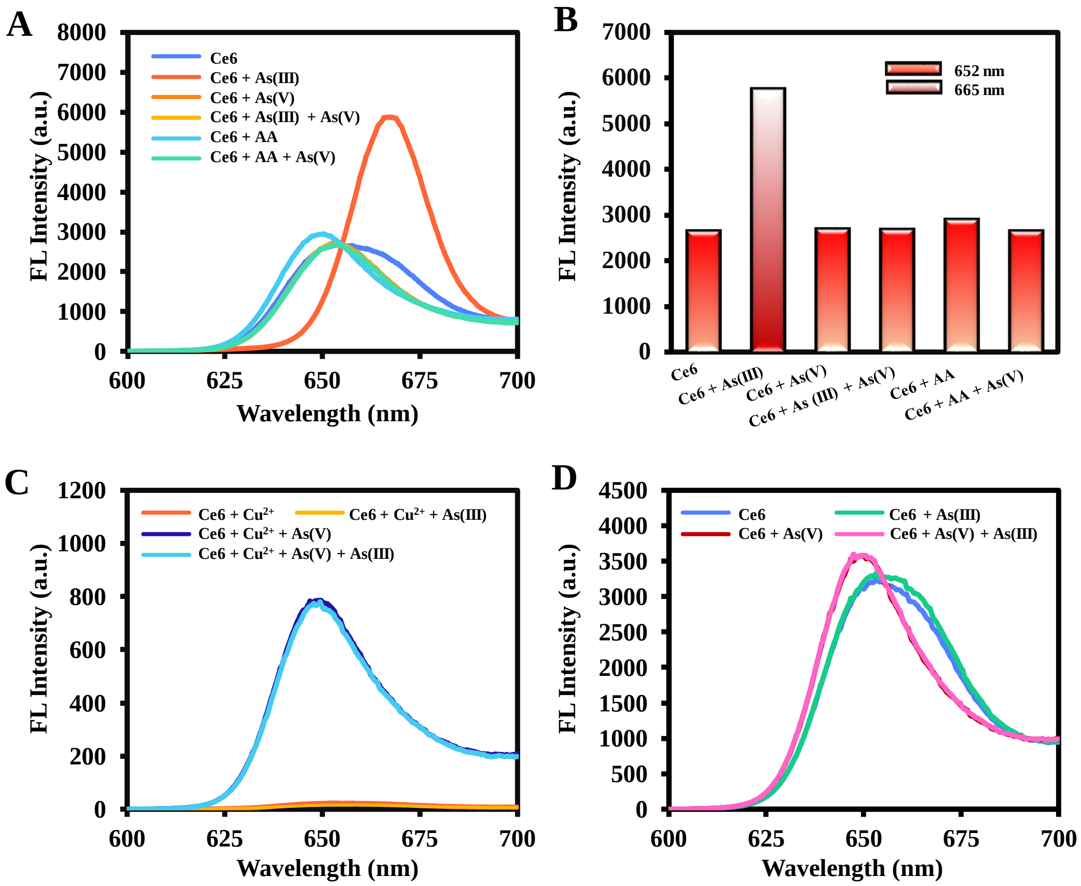

Figure S2. (**A**) The effect of different substances on the fluorescence spectrograms of Ce6 and (**B**) the corresponding histograms; (**C**) Influence of As(III) on the detection of As(V); (**D**) Influence of As(V) on the detection of As(III). Conditions: the volume of Ce6, Cu^{2+}, As(III), As(V), and AA are 200 µL, 100 µL, 100 µL, 100 µL, and 100 µL, respectively (If the substance is not present, make up the volume with ultrapure water to 1 mL); Ce6 concentration, 10 ppm in ethanol for a and B, 3.125 ppm in ethanol for C, 12.5 ppm in ethanol for D; Cu^{2+} concentration, 100 ppm in ultrapure water for C and D; As(V) concentration, 100 ppm in ultrapure water for A and B, 1 ppm in ultrapure water for C and D; AA concentration, 10 mM in ultrapure water for A and B; As(III) concentration, 100 ppm in ultrapure water for A and B, 1 ppm in ultrapure water for C and D; reaction temperature, 50 °C for A and B, 30 °C for C and D; reaction time, 5 min for A, B, C, and D; fluorescence spectra were recorded from 600–700 nm at an excitation wavelength of 400 nm (excitation/emission slit width: 2.5/10 nm, scan speed: 1200 nm/min, PMT Voltage: 400 V).

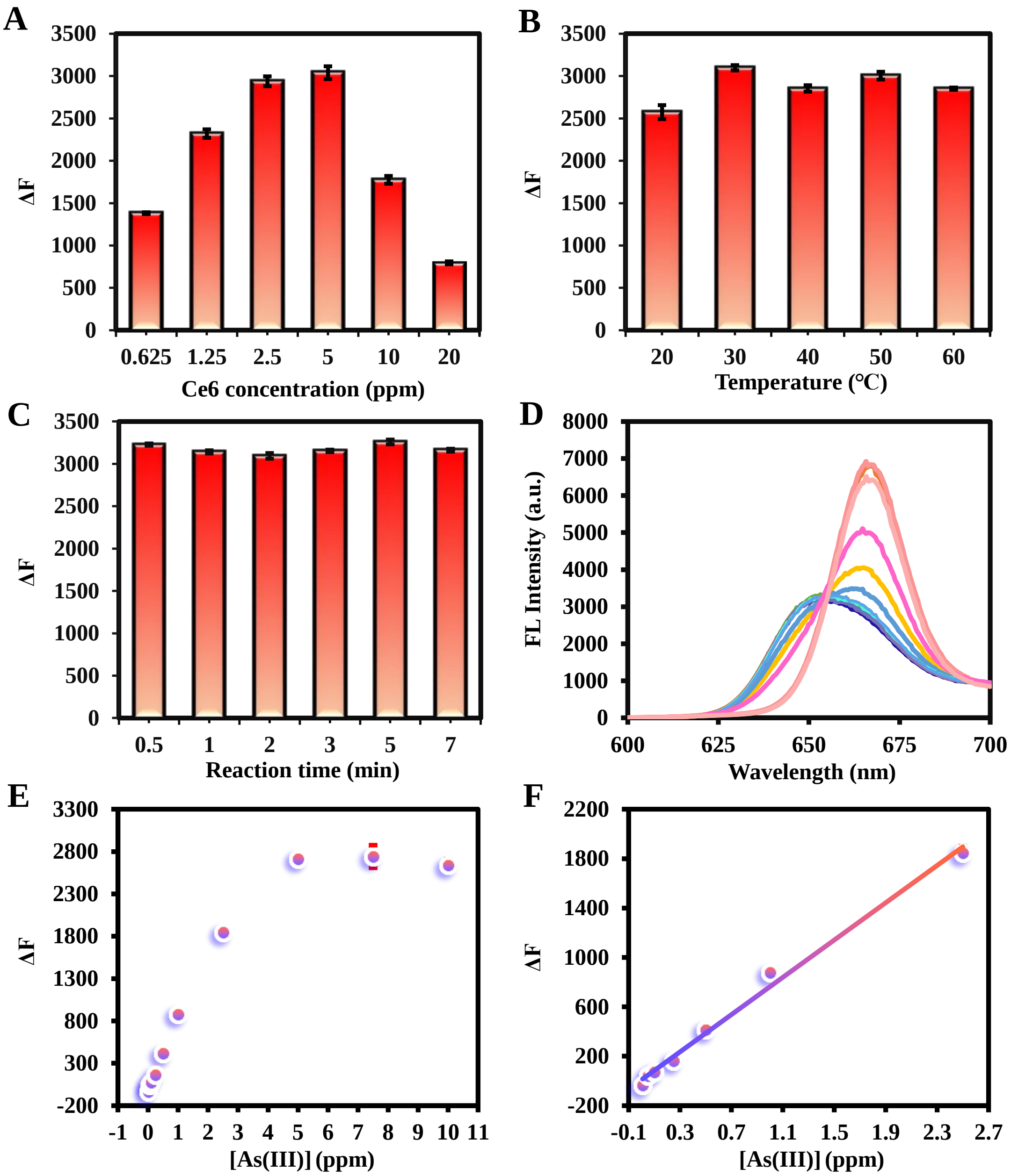

Figure S3. The effects of (**A**) Ce6 concentration, (**B**) reaction temperature, and (**C**) time on the fluorescence intensity for As(III) detection. (**D**) Fluorescence emission titration of Ce6 with different concentrations of As(III) at an excitation wavelength of 400 nm in ultrapure water and (**E** and **F**) linear plot of ΔF (at 665 nm) of As(III) concentration. Conditions: the volume of Ce6, ultrapure water, and As(III) are 200 μL, 700 μL, and 100 μL, respectively; Ce6 concentration, 12.5 ppm in ethanol for B, C, D, E, and F; As(III) concentration, 100 ppm in ultrapure water for A, B, and C; reaction temperature, 50 °C for A, 30 °C for C, D, E, and F; reaction time, 5 min for A and B, 1 min for D, E, and F; fluorescence spectra were recorded from 600–700 nm at an excitation wavelength of 400 nm (excitation/emission slit width: 2.5/10 nm, scan speed: 1200 nm/min, PMT Voltage: 400 V).

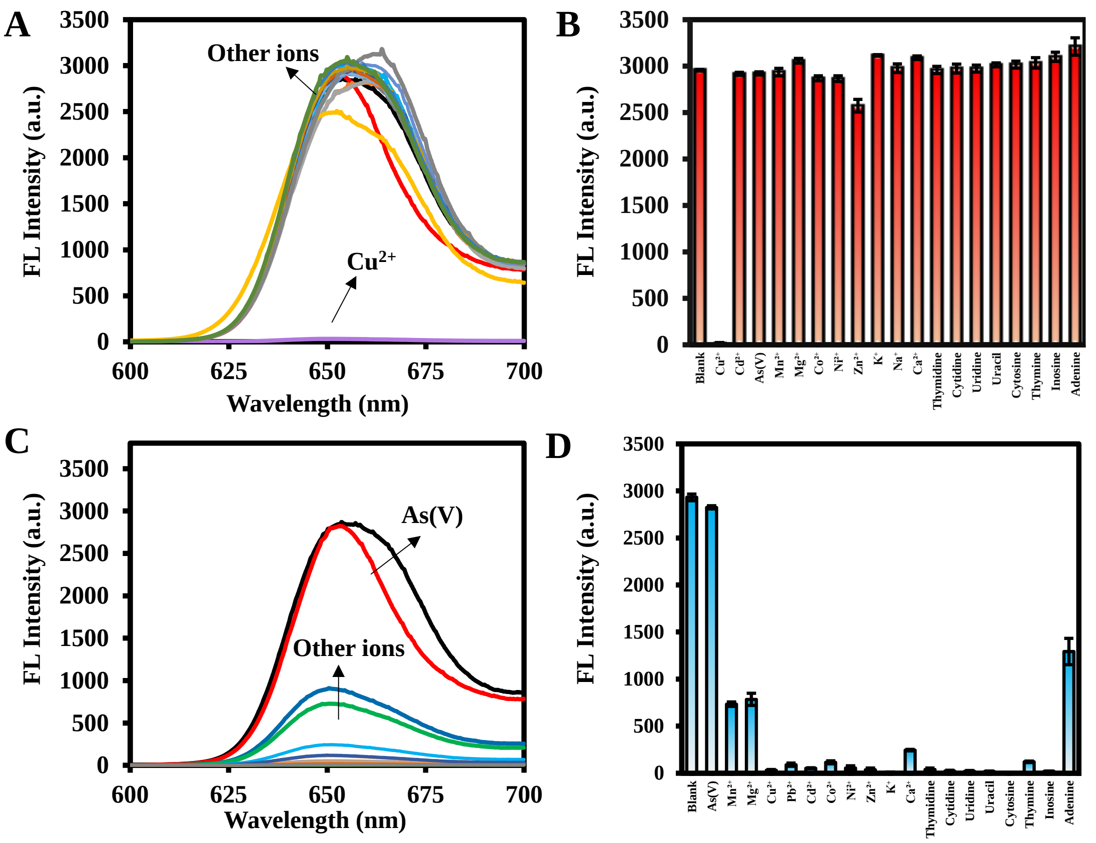

Figure S4. (**A** and **B**) Selectivity and (**C** and **D**) interference assays of Ce6 to 10 ppm of Cu^{2+} and other different anions. Conditions: the volume of Ce6, ultrapure water, and ions are 200 µL, 700 µL, and 100 µL, respectively for A and B, the volume of Ce6, ultrapure water, Cu^{2+} and ions are 200 µL, 600 µL, 100 µL, and 100 µL, respectively for C and D; Ce6 concentration, 6.25 ppm in ethanol; ions concentration, 100 ppm in ultrapure water; reaction temperature, 40 °C; reaction time, 7 min; fluorescence spectra were recorded from 600–700 nm at an excitation wavelength of 400 nm (excitation/emission slit width: 2.5/10 nm, scan speed: 1200 nm/min, PMT Voltage: 400 V).

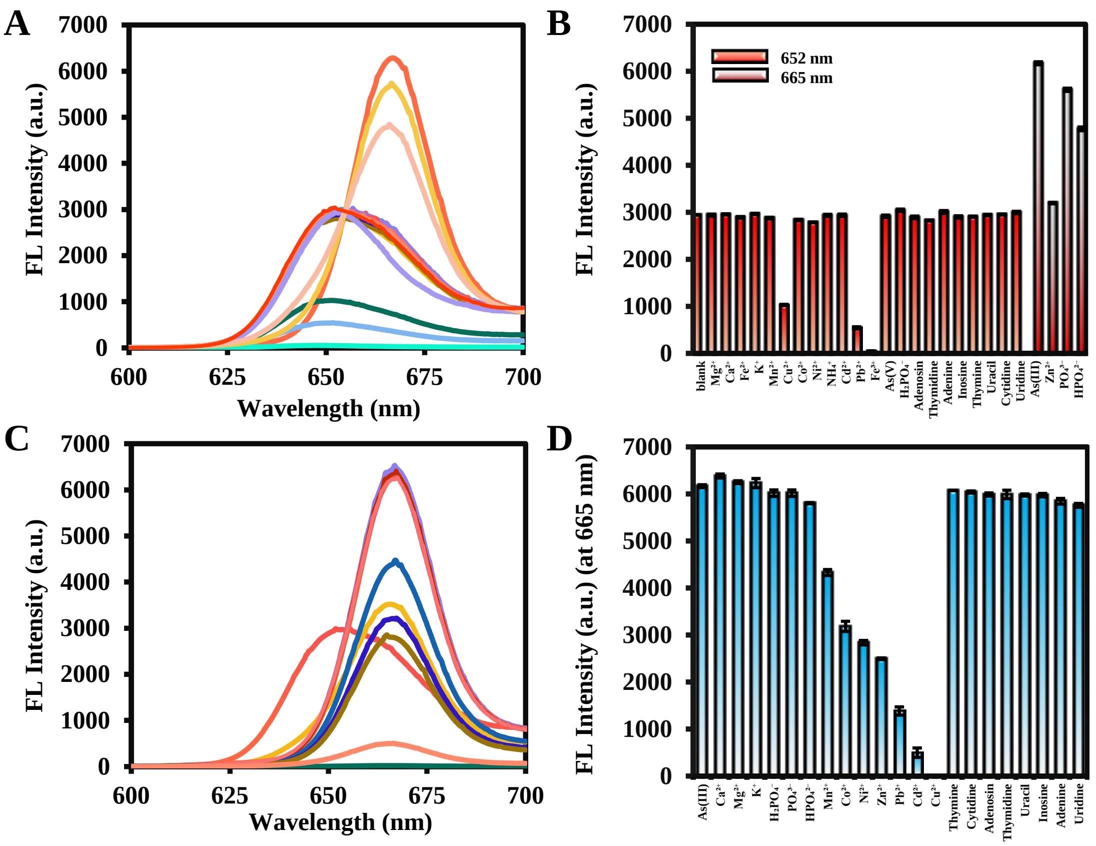

Figure S5. (**A** and **B**) Selectivity and (**C** and **D**) interference assays of Ce6 to 10 ppm of As(III) and other different anions. Conditions: the volume of Ce6, ultrapure water, and ions are 200 μL, 700 μL, and 100 μL, respectively for A and B, the volume of Ce6, ultrapure water, As(III) and ions are 200 μL, 600 μL, 100 μL, and 100 μL, respectively for C and D; Ce6 concentration, 12.5 ppm in ethanol; ions concentration, 100 ppm in ultrapure water; reaction temperature, 30 °C; reaction time, 1 min; fluorescence spectra were recorded from 600–700 nm at an excitation wavelength of 400 nm (excitation/emission slit width: 2.5/10 nm, scan speed: 1200 nm/min, PMT Voltage: 400 V).

Table S1 Detection of Cu^{2+} by fluorescent probes based on various small-molecule receptors.

Receptors	Detection mechanism	Linear range (μM)	LOD (μM)	Ref.
Probe **1**	Fluorescence on	1.25–6.3	0.676	[1]
Probe RBg	Fluorescence on	0.04–5.0	0.062	[2]
A5 fluorescence probe	Fluorescence on	2.5–40	0.11	[3]
HHPBA-BODIPY	Fluorescence off	0–1	0.35	[4]
FA	Fluorescence off	0.5–100	0.073	[5]
Imidazole-based probe **1**	Fluorescence off	–	3.2	[6]
Ce6	Fluorescence off	0.04–2.0	0.212	This work

Probe 1: benzimidazole-containing quinazoline based probe; probe RBg: a benzothiazole-oxanthracene structure is formed by incorporating amide bonds with the connecting group and the glyoxal identifying group; A5: a fluorescein derivative synthesized from fluorescein and hydrazine; FA: folic acid; HHPBA-BODIPY: based on boron-dipyrromethene (BODIPY), taking 2-hydroxy-N-(2-hydroxyphenyl)benzamide as recognition site.

Table S2 Determination of Cu^{2+} in real water and soil samples.

Samples	Added (ppm)	Founded (ppm)	Recovery [a] (%)	RSD (%) (n=3)
**Yun Lake**	0.00	–[b]		
	0.05	0.0550	110.1	2.2
	0.25	0.2365	94.6	2.0
	0.50	0.4797	95.9	2.7
**Jin Lake**	0.00	–		
	0.05	0.0469	93.8	3.8
	0.25	0.2224	89.0	3.4
	0.50	0.4742	94.9	0.2
**Yun Lake Soil**	0.00	–		
	0.05	0.0484	96.7	5.6
	0.25	0.2504	100.1	1.0
	0.50	0.5026	100.5	3.8
**Jin Lake Soil**	0.00	–		
	0.05	0.0485	97.1	4.1
	0.25	0.2599	104.0	1.1
	0.50	0.5519	110.4	6.3

[a] Recovery = (founded concentration-original concentration)/added concentration ×100%.

[b] The detection value is lower than the quantification limit of this method.

Table S3 Determination of As(III) in real water and soil samples.

Samples	Added (ppm)	Founded (ppm)	Recovery [a] (%)	RSD (%) (*n*=3)
**Yun Lake**	0.00	–[b]		
	0.50	0.4994	99.9	4.6
	1.00	1.1845	118.5	1.8
	2.50	2.3458	93.8	5.9
**Jin Lake**	0.00	–		
	0.50	0.4998	100.0	4.5
	1.00	1.1434	114.3	1.6
	2.50	2.4447	97.9	3.7
**Yun Lake Soil**	0.00	–		
	0.50	0.5239	104.8	2.6
	1.00	1.1355	113.6	2.6
	2.50	2.0558	82.2	5.1
**Jin Lake Soil**	0.00	–		
	0.50	0.4998	100.0	4.5
	1.00	1.1434	114.3	1.6
	2.50	2.4447	97.8	3.7

[a] Recovery = (founded concentration-original concentration)/added concentration ×100%.

[b] The detection value is lower than the quantification limit of this method.

References

1. Tang, L.; Zhou, P.; Zhong, K.; Hou, S. Fluorescence relay enhancement sequential recognition of Cu^{2+} and CN^- by a new quinazoline derivative. *Sens. Actuator B-Chem.* **2013**, *182*, 439–445, https://doi.org/10.1016/j.snb.2013.03.043.
2. Cheng, Z.; Jin, X.; Liu, Y.; Zheng, L.; He, H. An ESIPT-based fluorescent probe for aqueous cu^+ detection through strip, nanofiber and living cells. *Molecules* **2023**, *28*, 3725, https://doi.org/10.3390/molecules28093725.
3. Leng, X.; Wang, D.; Mi, Z.; Zhang, Y.; Yang, B.; Chen, F. Novel fluorescence probe toward Cu^{2+} based on fluorescein derivatives and its bioimaging in cells. *Biosensors-Basel* **2022**, *12*, 732, https://doi.org/10.3390/bios12090732.
4. Sun, R.; Wang, L.; Jiang, C.; Du, Z.; Chen, S.; Wu, W. A highly efficient BODIPY based turn-off fluorescent probe for detecting Cu^{2+}. *J. Fluoresc.* **2020**, *30*, 883–890, https://doi.org/10.1007/s10895-020-02544-9.
5. Zhang, Y.; Deng, Q.; Tang, C.; Zhang, M.; Huang, Z.; Cai, Z. Fluorescent folic acid-capped copper nanoclusters for the determination of rifampicin based on inner filter effect. *Spectroc. Acta Pt. A-Molec. Biomolec. Spectr.* **2023**, *286*, 121944, https://doi.org/10.1016/j.saa.2022.121944.
6. Okda, H.E.; El Sayed, S.; Otri, I.; Ferreira, R.C.M.; Costa, S.P.G.; Raposo, M.M.M.; Martínez-Máñez, R.; Sancenón, F. A simple and easy-to-prepare imidazole-based probe for the selective chromo-fluorogenic recognition of biothiols and Cu (II) in aqueous environments. *Dyes Pigment.* **2019**, *162*, 303–308, https://doi.org/10.1016/j.dyepig.2018.10.017.
